# Epidemiologic profile of *Staphylococcus aureus*-methicilin resistant (MRSA) bacterium in hospital-acquired-infection in neonatal-intensive-care-unit (ICU) from -2000 to 2010 analysis in general hospital

**DOI:** 10.1186/1753-6561-5-S6-P173

**Published:** 2011-06-29

**Authors:** LF Baqueiro-Freitas, CI Santos, F Ferreira

**Affiliations:** 1 Internal Medicine, University of Sao Paulo, Ribeirao Preto, Brazil; 2 Infection Control Service, Santa Lydia Hospital, Ribeirão Preto, Brazil; 3 Microbiology, Santa Lydia Hospital, Ribeirão Preto, Brazil

## Introduction / objectives

Awareness of epidemiologic profile of MRSA-hospital-acquired infection in neonatal-ICU might improve an early recognition of this infection.

Evaluating the clinical epidemiologic profile of hospital-acquired *Staphylococcus aureus*-bacterium infection in neonatal-ICU patients according to its methicilin sensibility.

## Methods

The infections cases were prospectively recorded for an eleven-year period from 2000 to 2010; the program used was EPI-INFO v 3.4.1.

## Results

31 strains of *Staphylococcus sp* were identified in some hospital-acquired-infections. 46.7% of *Staphylococcus aureus* were methicilin resistant. Bloodstream infection (BSI) was the most prevalent site of infection of MRSA (40%) as well as for methicilin-sensitive *Staphylococcus aureus* (MSSA) (62.5%). Symptoms of infection had began as early as 8.5 days and as late as 11 days (average time) from the admission date in methicilin-sensitive and methicilin-resistant *Staphylococcus aureus* cases respectively. The previous antibiotic therapy was more usual in MRSA cases (80%) than in MSSA (0%). The average weight of newborn infant was heavier in MSSA (2.222g) than MRSA (1626g). The frequency of death was higher in MRSA than MSSA (40% and 12,5% respectivelly). The average duration of stay was slightly longer in MRSA (24 days) than in MSSA (22.8 days).

## Conclusion

From that analysis we have pointed out an epidemiologic profile of MRSA-hospital infections in neonatal-ICU concerning its prevalence and others epidemiologic issues in order to prevent its increase and diffusion in neonatal ICU.

## Disclosure of interest

None declared.

